# Measuring Short-Term Outcomes Following Primary Total Hip Arthroplasty: A Value-Based Healthcare Approach

**DOI:** 10.3390/jcm14103310

**Published:** 2025-05-09

**Authors:** Panayiotis Christofilopoulos, Hugo Bothorel, Selina Bilger, Florian Rüter, Robyn Cody, Karl Stoffel

**Affiliations:** 1 Department of Trauma and Orthopaedic Surgery, La Tour Hospital, 1217 Meyrin, Switzerland; panayiotis.christofilopoulos@latour.ch; 2 Research Department, La Tour Hospital, 1217 Meyrin, Switzerland; 3 Department of Quality Management and Value-Based Healthcare, University Hospital Basel, 4031 Basel, Switzerland; selinasimone.bilger@usb.ch (S.B.); florian.rueter@usb.ch (F.R.); robyn.cody@usb.ch (R.C.); 4 Department of Trauma and Orthopaedic Surgery, University Hospital Basel, 4031 Basel, Switzerland; karl.stoffel@usb.ch; 5 University of Basel, 4001 Basel, Switzerland

**Keywords:** value-based healthcare, VBHC, total hip replacement, total hip arthroplasty

## Abstract

**Background/Objectives:** Total hip arthroplasty (THA) is a highly effective treatment for end-stage hip disease, but the increasing volume of procedures demands a focus on value-based healthcare (VBHC) to ensure optimal outcomes. This study proposes a novel approach to evaluate the value delivered by THA using direct costs and patient-reported outcome measures (PROMs). **Methods**: This retrospective cohort study included patients undergoing primary THA for unilateral osteoarthritis at two hospitals between 2018 and 2021. PROMs specific to hip osteoarthritis were assessed preoperatively and in the second postoperative year. The delivered *quality* was calculated using PROM results in comparison with the minimal clinically important difference (MCID) and patient acceptable symptom state (PASS) thresholds. The associated *cost* was defined as direct THA expenses in comparison with the median direct costs of the cohort series, and the *value* was calculated as the ratio of *quality* over *cost*. A multivariable linear regression was performed to identify the factors associated with the THA *value*. **Results**: Among 224 patients (70 ± 10 years, 46% males), THA was of satisfactory *value* (≥1.0) for 82%. The THA *value* was lower for patients of female sex (β −0.27, *p* = 0.047), with higher preoperative PROMs (β −0.36, *p* < 0.001), previous contralateral THA (β −0.36, *p* = 0.049), or ipsilateral hip surgery (β −1.41, *p* < 0.001) with custom (β −0.76, *p* = 0.011) or fully cemented (β −0.83, *p* = 0.021) implants. **Conclusions**: The proposed methodology effectively assessed the THA value, revealing satisfactory outcomes for most patients but also identifying areas for improvement. These findings emphasize the need for risk-adjusted VBHC models to enhance equity and efficiency in arthroplasty care.

## 1. Introduction

Since its introduction in the 1960s, total hip arthroplasty (THA) has become a surgical procedure that satisfies a high proportion of patients with end-stage hip disease (>85%), [1] and lasts at least 25 years in more than half of the cases [2]. Over the past decades, the rate of hip replacement has been steadily increasing, driven by an increased demand for improved mobility and quality of life in an ageing population [3,4]. This rise is not uniform across countries and highlights significant international differences. Data on procedures performed in 2021 show that Switzerland had the highest rate of hip replacement (323/100,000 population) among OECD countries (172/100,000 in average) (OECD Health Statistics, Supplementary Materials S1) [5]. Differences in population structure may explain part of this variation, though significant disparities persist after age standardization [6]. While such variations cannot be directly attributed to the question of inadequate care, they do highlight the need to assess whether all patients truly benefit from surgery. If no action is taken, hip replacements that do not benefit patients will soon become a significant burden, not only for the patients themselves but also for healthcare systems [4].

The disruptive concept of value-based healthcare (VBHC) has emerged to move the current system towards a sustainable, patient-centered model that optimizes both costs and health outcomes [7]. On the one hand, such a model motivates healthcare institutions to optimize their resources as they are confronted with cost escalation, particularly those associated with continuous technological advances. On the other hand, it also encourages them to evaluate THA results according to the health outcomes that matter the most to the patients in their daily lives. This can be effectively done using specific patient-reported outcome measures (PROMs), covering domains relevant to hip osteoarthritis (OA), such as pain and functional activities. However, in the absence of any real consensus, a wide variety of PROMs are being used, which initially makes it difficult to benchmark and aggregate the results at a higher level than the institution itself (such as inter-institutional, regional, or national levels).

Various PROM-specific thresholds have been newly developed to help clinicians and researchers to assess the clinical relevance of patient’s evolution following surgery, such as the minimum clinically important difference (MCID), substantial clinical benefit (SCB), and patient acceptable symptom state (PASS). Such thresholds provide a standardized way of analyzing PROMs and have, therefore, become of great interest in VBHC [8]. Few authors have already worked on a calculation method to assess the value delivered by a total joint replacement, interpreting PROMs with either the MCID or SCB thresholds [9,10]. However, reliance on either of these thresholds alone can be misleading. Several VBHC experts now recommend using the MCID and PASS thresholds in relation to each other, [8] as the true objective is to make the patient feel better and feel good after surgery [11]. The objective of this study was to evaluate the value (*value*) delivered by primary THA across two institutions, using an innovative patient-level metric that integrates PROMs relative to the MCID and PASS thresholds (*quality*), as well as direct cost data (*cost*). Furthermore, the authors aimed to evaluate the factors associated with greater *quality*, *cost*, and *value*.

## 2. Materials and Methods

This retrospective cohort study comprised patients operated on at La Tour Hospital (Meyrin, Switzerland) and University Hospital Basel (USB) (Basel, Switzerland) for primary THA between January 2018 and December 2021. The patients were included if they (1) were coded in the diagnosis-related group (DRG): “Implanting, replacing or revising a hip endoprosthesis” and recorded in the Swiss national registry for primary hip replacement (SIRIS), with primary unilateral osteoarthritis as the principal diagnosis; (2) did not refuse the use of their data for research purposes. The patients were excluded if (1) the PROM collection system was not used to assess the preoperative status of patients; (2) preoperative PROMs were not collected in the two months preceding the surgery; (3) postoperative PROMs were not collected in the second year following the surgery (between 12 ± 2 and 24 ± 2 months); (4) in any case of unavailable/unreliable cost data. It is worth mentioning that the patients who experienced a complication, revision, or contralateral THA prior to PROM collection were not excluded. This study was approved by the ethics committee of Geneva (CCER 2023-00693), and the requirement for written informed consent was waived for the patients who could not be contacted by the investigators and had not previously refused the use of their data for research purposes.

The patient characteristics recorded included their age, sex, body mass index (BMI), operated side, previous hip surgery, insurance type, American Society of Anesthesiologists (ASA) score, and Charnley grade (A: contralateral hip not affected; B1: contralateral hip affected but untreated; B2: existing THA on the contralateral hip; C: functional limitations due to other joint involvement or systemic diseases). The surgical characteristics recorded included the acetabular implant details (single/dual mobility cup, standard/custom-made stem), patient installation (dorsal/lateral decubitus), surgical approach, use of extension table, THA type (uncemented/hybrid/cemented/), and length of stay (LOS). Both the patient and surgical characteristics were extracted from the SIRIS registry using a standardized procedure by trained personnel. The patients were assessed pre- and postoperatively, either by a single PROM (the modified Harris Hip Score, mHHS) [12] or by a combination of two PROMs (the pain on a visual analogue scale [pVAS] and the Hip disability and Osteoarthritis Outcome Score—Physical function Short form [HOOS-PS]). The use of different PROMs (mHHS vs. HOOS-PS + pVAS) reflects institutional differences in data collection protocols. To address this variability, we employed a methodology based on PROM-specific thresholds (MCID and PASS) and domain weighting, allowing for more meaningful comparisons across the datasets. The mHHS ranged from 0 (worst) to 100 (best) and assessed both function (52 points) and pain (48 points). The MCID and PASS thresholds used for the mHHS were, respectively, 8 points [13,14] and 85 points [15]. The pVAS used in this study was reverse-coded, such that 0 indicated the maximum pain and 100 indicated no pain, while the HOOS-PS ranged from 0 (worst) to 100 (best) and assessed function only. The MCID and PASS thresholds used for the pVAS were, respectively, 20 points [16,17,18] and ≥ 65 points [19] after rescaling. The MCID and PASS thresholds used for the HOOS-PS were, respectively, 10 points [20] and 88 points [19]. A composite PROM was then created using both the pVAS and the HOOS-PS so that the pain and function domains were weighted equally with those of the mHHS: pVAS × 0.48 + HOOS-PS × 0.52. The adjusted MCID and PASS thresholds for this composite PROM were calculated to be 15 points and 77 points, respectively.

### 2.1. Quality Assessment

The quality delivered by THA was assessed using (1) the extent of PROM improvement (P_ΔPROM_) following the surgery in comparison with the MCID and (2) using the postoperative PROM (P_PostPROM_) in comparison with the PASS. Quality was forced to 0 in case of an implant revision or negative pre- to postoperative PROM changes [10]. The patients were stratified into three responder groups based on a method inspired by Bernstein et al. [8] which was adapted by the authors. The aim was to give equal weight (0.5) to PROM improvement relative to the MCID and to the postoperative PROM level relative to the PASS. A specific calculation method for the quality assessment was applied to each group accordingly:

- Nonresponders (*Did not achieve the MCID*):

(1) $$\mathrm{quality}=0.5\;\times \;\frac{P_{\Delta \mathrm{PROM}}}{\mathrm{MCID}}$$

- Moderate responders (*Achieved the MCID but did not reach the PASS*):

(2) $$\mathrm{quality}=0.5+0.5\;\times \;\;\frac{P_{\mathrm{PostPROM}}}{\mathrm{PASS}}$$

- High responders (*Achieved the MCID and reached the PASS*):

(3) $$\mathrm{quality}=0.5\;\times \;\frac{P_{\Delta \mathrm{PROM}}}{\mathrm{MCID}}+0.5\;\times \;\frac{P_{\mathrm{PostPROM}}}{\mathrm{PASS}}$$

### 2.2. Cost Assessment

In this study, the THA cost (P_Directcost_) was defined as the direct cost related to the surgical procedure (medical material, implant, and medicine costs only) and was expressed in Swiss francs (CHF). These data were exported from the management accounting REKOLE^®^ analyses that were performed annually at each institution. Given the absence of a standard reference for the THA direct costs in Switzerland, the authors decided to consider the median direct costs of the studied THA series as the theoretical target (T_Directcost_).

(4) $$\mathrm{cost}=\frac{P_{\mathrm{Directcost}}}{T_{\mathrm{Directcost}}}$$

### 2.3. Value Assessment

The delivered *value* was then calculated for each THA using both *quality* and *cost* assessments, and it was considered as satisfactory if ≥1.0:

(5) $$\mathrm{value}=\frac{\mathrm{quality}}{\mathrm{cost}}$$

### 2.4. Statistical Analyses

Descriptive statistics were used to summarize the data. The normality of continuous variable distributions was assessed and determined using a histogram, quantile–quantile (QQ) plot, and Shapiro–Wilk test. Continuous variables were reported as mean ± standard deviation (if normally distributed) or median [interquartile range (IQR)] (if not normally distributed), and categorical data were reported as proportions. Comparisons of continuous data between the preoperative status and the postoperative status were conducted using paired Student *t*-tests (if normally distributed) or Wilcoxon signed-rank tests (if not normally distributed). Comparisons of continuous data between the independent groups of patients were conducted using unpaired Student *t*-tests (if normally distributed) or Wilcoxon rank sum tests (if not normally distributed). The correlation between *quality* and *cost* was analyzed using the Pearson’s coefficient and interpreted as negligible (0.00–0.10), weak (0.10–0.39), moderate (0.40–0.69), strong (0.70–0.89), or very strong (0.90–1.00). Multivariable linear regression models were performed to identify which factors (age, sex, BMI, previous hip surgery, Charnley grade, ASA score, institution, custom/standard implant, cemented/uncemented THA, length of stay, and year of intervention) were independently associated with the THA *quality*, *cost*, and *value*. The selection of potential predictors was theory-driven, based on their clinical relevance. To ensure an adequate statistical power for this analysis, a rule of 10 subjects per potential predictor was respected. We estimated that a maximum of 20 potential predictors would be included in the model; therefore, this analysis required a minimum of 200 patients with complete data in the model. Assumptions for linearity, homoskedasticity, residual normality, and the absence of multicollinearity among the independent variables were also verified. Collinearity was assessed using the Variance Inflation Factor (VIF) and was deemed acceptable if the maximum VIF did not exceed 4.0. An important multicollinearity led to covariate deletion (e.g., surgical approach) or variables merging (e.g., institution and implant type). The association between each independent variable and the outcome studied (*quality*/*cost*/*value*) was expressed with a regression coefficient and the 95% confidence interval (β, [95%CI]). Regression coefficients for the age and preoperative PROMs in the multivariable linear models were adjusted for a 10-unit increment. Statistical analyses were performed using R version 3.6.2 (R Foundation for Statistical Computing, Vienna, Austria), and *p*-values < 0.05 were considered statistically significant.

## 3. Results

**Figure 1:**
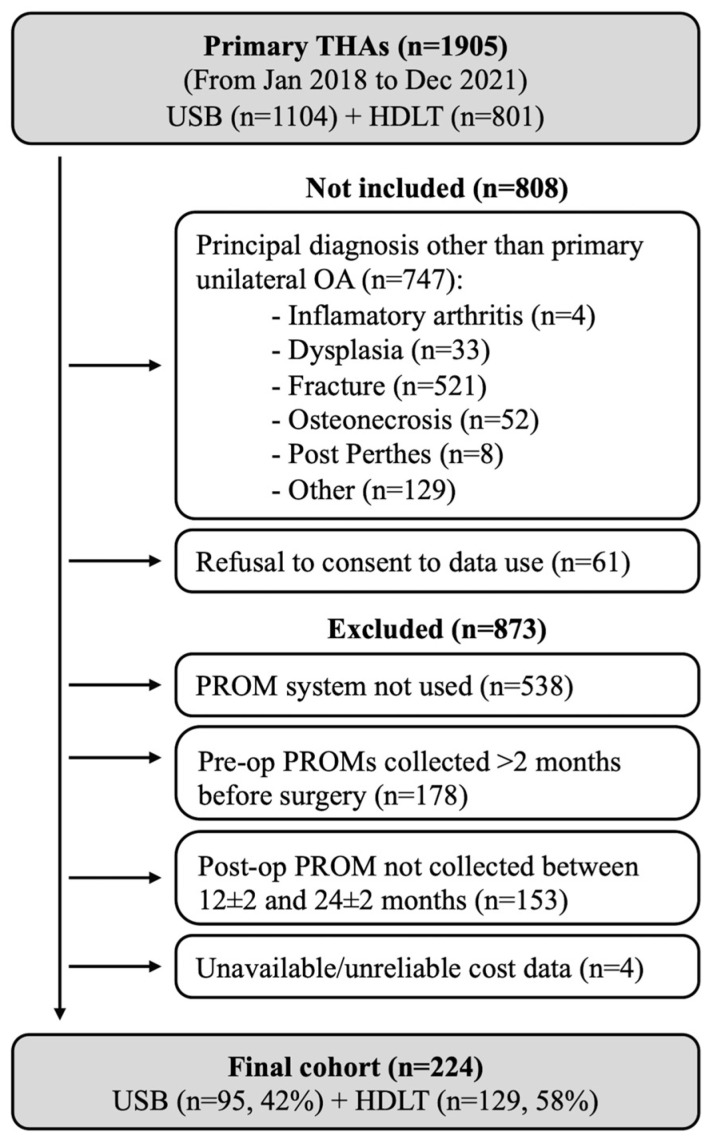
Study flowchart.

A total of 1905 THAs were performed in the inclusion period, either at USB (*n* = 1104, 58%) or La Tour Hospital (*n* = 801, 42%). After applying the inclusion and exclusion criteria, the study cohort finally comprised 224 patients aged 70 ± 10 years at the time of index surgery (46% males) (Table 1, Figure 1). The patients were operated on by nine different surgeons, either at La Tour Hospital (*n* = 129, 58%) or USB (*n* = 95, 42%). Most of the patients received a single mobility THA (*n* = 220, 98%) without custom-made implants (*n* = 208, 93%).

At the postoperative follow-up (median 12 months [IQR, 11 to 15]), one patient (0.4%) had an implant revision. Three patients (1%) underwent contralateral THA during the follow-up period, but this did not substantially affect the postoperative PROMs related to the first THA (100 pts, 100 pts, and 80 pts). The PROMs significantly increased from 57 ± 17 preoperatively to 91 ± 15 postoperatively (*p* < 0.001) (Table 2). Nineteen patients (8%) did not report a PROM improvement above the MCID (nonresponders rate), 21 (9%) improved above the MCID but did not reach the PASS postoperatively (moderate responders), and 184 (82%) achieved both (high responders) (Table 2).

According to the defined calculation method, the *quality* of the care delivered to the patients was 2.06 ± 0.94. A multivariable linear regression revealed that the delivered *quality* decreased with higher preoperative PROMs (−0.35 [−0.43 to −0.27]) and was lower for patients with previous hip surgery (−0.96 [−1.56 to −0.37]); patients operated on with a fully cemented THA (−0.71 [−1.33 to −0.09]), compared to an uncemented THA; patients operated on in 2020 (−0.57 [−0.97 to −0.16]) and in 2021 (−0.46 [−0.86 to −0.07]), compared to 2018; and patients operated on at USB (−0.80 [−1.15 to −0.44]) (Table 3). The *quality* also tended to be lower for female patients (−0.21 [−0.45 to 0.02]) and for patients with a B2 Charnley grade (−0.31 [−0.63 to 0.01]), compared to grade A.

The direct cost per THA was CHF 5247 [IQR, 4125 to 6362], which comprised CHF 4958 [IQR, 3921 to 6014] of medical materials and implants. The direct costs decreased from CHF 6777 [IQR, 6443 to 7395] in 2018 to CHF 5723 [IQR, 4322 to 6062] in 2020 and to CHF 4405 [IQR, 3875 to 5013] in 2021, corresponding to a respective decrease of 16% and 35%, approximately. According to the defined calculation method, the *cost* per case was 1.0 [IQR, 0.79 to 1.21]. A multivariable linear regression revealed that the *cost* was higher for patients operated on with custom-made implants (0.81 [0.74 to 0.89]) and at La Tour hospital (0.24 [0.19 to 0.29]) (Table 3). The *costs* were lower for operations performed in 2020 (−0.20 [−0.26 to −0.14]) and 2021 (−0.30 [−0.36 to −0.25]), compared to 2018, and were slightly lower for hybrid THA (−0.06 [−0.11 to −0.02]) compared to uncemented THA (Table 3).

**Figure 2:**
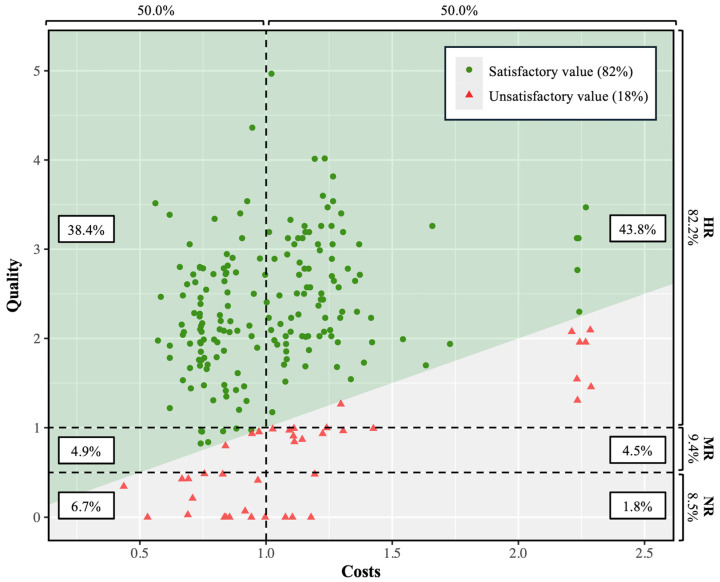
Scatter plot illustrating cost versus quality measures with patient delivered value. (HR, high responders; MR, moderate responders; NR, nonresponders).

In accordance with the defined calculation method, the *value* delivered by THA was 2.10 ± 1.08, which was ≥1.0 in 184 cases (82%) (Figure 2). The correlation between the *cost* and the delivered *quality* was weak (r = 0.18 [0.05 to 0.30]). A multivariable linear regression revealed that the THA *value* decreased with higher preoperative PROMs (−0.36 [−0.45 to −0.26]) and was lower for patients with previous hip surgery (−1.41 [−2.09 to −0.74]); custom-made implants (−0.76 [−1.35 to −0.18]); a B2 Charnley grade (−0.36 [−0.73 to −0.00]), compared to grade A; and fully cemented implants (−0.83 [−1.53 to −0.12]), compared to uncemented THA; as well as for female patients (−0.27 [−0.53 to −0.00]) (Table 3).

## 4. Discussion

In the context of escalating costs and volumes of orthopedic treatments, healthcare systems are currently under pressure to optimize both their resources and their quality of care. The authors, therefore, proposed a standardized approach to evaluate the value delivered by THA at a multicentric level based on direct costs and PROMs following surgery. The main finding of the study was that the THA value was satisfactory in 82% of the patients who were operated on, which leaves some room for improvement. The delivered *value* was lower for the patients with higher preoperative PROMs, previous hip surgery, contralateral THA, custom-made implants, and cemented implants, as well as for female patients. Such information emphasizes the importance of risk adjustment in VBHC models that aim to assess or compare the value delivered at an institutional or multicenter level.

Once both the MCID and PASS thresholds are reached, the delivered *quality* (as calculated in the proposed equation) is mainly driven by the PROMs’ improvement following surgery. Therefore, the THA was of greater *value* for the patients who started from a worse preoperative health status with more room for improvement [21]. However, this finding should be interpreted with caution, given this study included direct costs and short-term outcomes only. Selecting patients with a low preoperative health status for surgery can lead to an increase in costly complications and suboptimal outcomes at longer follow-ups. In addition, other studies should determine the real impact of delaying surgery, considering the costs of injections and medications, as well as of other non-operative modalities [21], and evaluate the most appropriate THA timing in the course of OA to optimize its cost-effectiveness in the long term [22].

Other factors associated with a lower THA *value* and *quality* due to lower PROM improvements included previous ipsilateral hip surgery and contralateral THA. These findings are consistent with the results recently reported by Peters et al. using the Dutch arthroplasty register [23] and by the Cleveland clinic adult reconstruction research group [24]. Fully cemented THA, which corresponded to 4% of the present THA series, also delivered lower *quality* and less *value* compared to uncemented THA. It might be due to cofounding factors that were not included in our statistical analyses (e.g., radiological data) but is consistent with a registry study showing that patients with cemented THA have less improvement in EQ-5D, pain, and satisfaction at the one year follow-up [25]. Uncemented THA is now considered a safe option in the elderly population [26,27], with comparable or better survivorship at the 20 years follow-up [28,29]. The current trend towards the increased use of uncemented THAs (representing more than 87% of all THAs for primary hip OA in Switzerland [30]), therefore, appears to be beneficial for both patients and the healthcare system.

It has been already identified that female patients feel greater improvements after THA [23]. This can be explained by the fact that they tend to have lower functional scores and more pain prior to surgery [31,32], giving them more room for improvement. However, when adjusted for preoperative PROMs, advanced statistical analyses reveal lower outcomes for female patients in the short term [32,33,34,35]. This is consistent with our results, which illustrated lower delivered *quality and value* for female patients, regardless of their preoperative and surgical characteristics. This highlights the need to further investigate the underlying causes (e.g., greater osteoporosis due to natural hormonal imbalances), while ensuring that such differences are included in risk-stratification models to ensure equitable arthroplasty care [36].

It is important to underline that both hospitals provided a comparable THA *value* to patients, even though they differed in terms of the *cost* and the delivered *quality*. Our findings suggest that both public and private hospitals may be capable of achieving comparable values under a VBHC framework, although this requires further validation using standardized metrics. The observed differences in the quality and the cost between La Tour Hospital and USB may reflect organizational factors, such as the hospital type (private vs. public), differences in perioperative care protocols, or the type of/access to rehabilitation. These elements were not systematically assessed in this study but could partly explain the variability in outcomes and warrant further investigation in future prospective studies.

Furthermore, significant efforts have been made over the last three years of inclusion to reduce direct costs by around 30%. It shows the continuous efforts made by both institutions but also highlights the importance of involving device manufacturers as key stakeholders in VBHC models that actively participate in such negotiations. These efforts could, for example, have compensated for the fact that the *quality* declined in the last years of inclusion (especially in 2020 compared to 2018). Given that the *cost* and the *quality* correlated weakly only, PROM worsening should not be related to direct cost reductions. The COVID-19 pandemic (2020–2021 in Switzerland), however, may have had an impact on the patients who were operated on for different reasons, including a limited access to physical therapy, the inability to exercise, and no face-to-face follow-up [37].

At the postoperative follow-up, custom implants offered patients a comparable *quality* to standard implants, but at a higher *cost*. This ultimately resulted in a lower delivered *value*. Such results were to be expected given the short follow-up of the study and the fact that custom stems are more likely to be implanted in patients with higher expectations and/or distorted anatomy. It is worth noting that the satisfactory clinical and radiological outcomes of custom implants have already been reported in the long term for such patients [38,39,40]. This motivates us to keep using them for some patients to optimize implant stability and the restoration of the native hip mechanics. The authors decided not to exclude these cases from the study in order to emphasize that innovation should not be discouraged in VBHC but rather continuously evaluated to ensure that the benefits outweigh the higher initial costs involved. Given that hospital costs and reimbursements can differ in a DRG-based reimbursement system, it also highlights the importance of working closely with payers/insurers to build collective initiatives and ensure that all parties benefit from such initiatives.

The proposed “*value* = *quality*/*cost*” model has promising applications beyond theoretical interest. For clinicians, it could serve as a decision support tool to identify the patient subgroups likely to experience lower value—such as those with previous surgery, higher preoperative PROMs, or certain implant types—and allow for the better management of patient expectations, the optimization of care pathways, or even the reconsideration of surgical timing. From a systemic perspective, this metric could assist hospital administrators and health insurers in benchmarking their institutional performance, supporting outcome-based reimbursement strategies, or promoting cost-effectiveness without compromising patient-centered outcomes.

### Limitations

This study has several limitations. Firstly, our analyses were performed at a short follow-up, which ranged from 12 ± 2 months to 24 ± 2 months. Even though a long-term analysis needs to be performed to fully determine the value of THA, evaluating THA outcomes shortly after surgery can be of importance given that poor PROMs at this time-point represent a risk factor for revision within the subsequent years [41]. Furthermore, the choice of this follow-up window to increase patient inclusion is supported by the fact that 1-year and 2-year PROMs after THA are comparable [42]. Secondly, the PROM collection was challenging due to the advanced age of the patients and the early-stage implementation of the PROM system, which was occasionally unstable at the time. We did not use any imputation techniques for missing data and did a complete case analysis. However, the excluded cases (*n* = 873) were comparable to the included ones (*n* = 224) in terms of the age and sex distributions, with no clinically relevant differences (age: 67 ± 13 vs. 70 ± 10, *p* < 0.001; women: 54.0% vs. 54.5%, *p* = 0.950). Furthermore, among the excluded cases with available preoperative PROMs within 2 months of surgery (*n* = 153), the PROM levels were comparable (58.6 ± 15.9 vs. 57.3 ± 17.2, *p* = 0.580), limiting the potential bias and supporting the study’s validity and generalizability. Thirdly, although the composite score provides a comprehensive assessment of the outcome, its lack of formal validation highlights the need for further methodological research. Furthermore, newly developed PROMs, such as the Forgotten Joint Score, could have been used to better assess the additional benefits of custom implants. However, such PROMs were not collected in the clinical routine at that time. Fourthly, the current DRG-based reimbursement system prevents us from defining external references for costs analyses. Therefore, we used the median costs of the studied THA cohort as a theoretical reference. Furthermore, indirect costs were not considered in the evaluation given they can comprise costs that are not related to the studied surgery per se. This led to the use of the direct cost of surgery (material and drugs only) for a more objective and unbiased assessment. Fifth, we were unable to calculate the MCID and PASS thresholds for each PROM using our own dataset due to the limited sample size. Therefore, we relied on thresholds reported in previous studies involving comparable patient populations, in order to approximate them as appropriately as possible. Finally, this study did not include radiological data (e.g., the degree of osteoarthritis degeneration, hip dysplasia, or femoral head deformity) and information on the patients’ preoperative socioeconomic, mental, and emotional health status. These factors are known to affect PROMs, and their omission may have introduced unmeasured confounding into the estimation of the THA *value*. Future studies should aim to include standardized radiographic grading and validated psychosocial questionnaires (e.g., the SF-12 Mental Component Score) to provide a more comprehensive and risk-adjusted understanding of the determinants of THA outcomes.

## 5. Conclusions

This study described a new methodology that evaluates short-term THA outcomes at the multicenter level and follows a VBHC approach. Our results revealed that the THA *value* was satisfactory for 82% of the operated cases, which leaves some room for improvement. The delivered value differed according to several patient characteristics, such as their sex, history of previous surgery, or Charnley class, thus emphasizing the need for risk-stratification models to ensure equitable arthroplasty care in the future.

## Figures and Tables

**Table 1:** Patient and hospitalization characteristics.

	Total (*n* = 224 Patients)	
**Patient characteristics**		
Age, mean (SD)	70	(10)
BMI, mean (SD)	26	(4)
Male sex, N (%)	102	(46)
Previous hip surgery, N (%) *	8	(4)
Charnley grade, N (%) **		
A	119	(53)
B1	60	(27)
B2	33	(15)
C	2	(1)
ASA score, N (%)		
1	30	(13)
2	133	(59)
3	59	(26)
4	2	(1)
**Hospitalization characteristics**		
LOS (days), mean (SD)	4	(2)
Surgical approach, N (%)		
Anterior	132	(59)
Anterolateral	88	(39)
Posterior	4	(2)
Custom-made THA, N (%)	16	(7)
Cementation type, N (%)		
Uncemented	141	(63)
Hybrid ***	75	(33)
Fully cemented	8	(4)

BMI, body mass index; ASA, American Society of Anesthesiologists; LOS, length of stay; THA, total hip arthroplasty; SD, standard deviation. * Hip arthroscopy (*n* = 3), femoral osteosynthesis (*n* = 2), femoral osteotomy (*n* = 2), dynamic hip crew (*n* = 1). ** missing data (*n* = 10, 4%). *** AC uncemented and FE cemented.

**Table 2:** PROM improvement.

	Total (*n* = 224 Patients)	
Preoperative PROM, mean (SD)	57	(17)
Postoperative PROM, mean (SD)	91	(15)
≥PASS, N (%)	188	(84)
PROM improvement, mean (SD)	34	(18)
≥MCID, N (%)	206	(92)
PROM interpretation, N (%)		
Nonresponders (<MCID)	19	(8)
Moderate responders (≥MCID AND <PASS)	21	(9)
High responders (≥MCID AND ≥PASS)	184	(82)

PROM, patient-reported outcome measure; SD, standard deviation; PASS, patient acceptable symptom state; MCID, minimal clinically important difference.

**Table 3:** Multivariable linear regression analysis of THA *quality*, *cost*, and *value*.

		***Quality***				***Cost***				***Value***		
		**β**	**95% C.I.**	***p***		**β**	**95% C.I.**	***p***		**β**	**95% C.I.**	***p***
Age (yrs) *		0.08	(−0.07 to 0.22)	0.293		−0.01	(−0.03 to 0.01)	0.443		0.08	(−0.08 to 0.24)	0.311
BMI		0.00	(−0.03 to 0.02)	0.765		0.00	(−0.00 to 0.01)	0.123		−0.01	(−0.04 to 0.02)	0.451
Male sex		0.21	(−0.02 to 0.45)	0.076		−0.01	(−0.04 to 0.02)	0.576		0.27	(0.00 to 0.53)	0.047
Preoperative PROM *		−0.35	(−0.43 to −0.27)	<0.001		0.00	(−0.01 to 0.01)	0.957		−0.36	(−0.45 to −0.26)	<0.001
Previous hip surgery		−0.96	(−1.56 to 0.37)	0.002		0.05	(−0.03 to 0.14)	0.221		−1.41	(−2.09 to −0.74)	<0.001
ASA score												
1		REF				REF				REF		
2		−0.18	(−0.53 to 0.17)	0.308		0.02	(−0.03 to 0.07)	0.392		−0.04	(−0.44 to 0.36)	0.851
3		−0.39	(−0.80 to 0.03)	0.066		0.03	(−0.03 to 0.09)	0.361		−0.27	(−0.74 to 0.20)	0.263
4		−0.55	(−1.72 to 0.61)	0.350		0.12	(−0.05 to 0.29)	0.152		−0.89	(−2.22 to 0.43)	0.185
Charnley grade												
A		REF				REF				REF		
B1		−0.04	(−0.30 to 0.21)	0.737		0.02	(−0.01 to 0.06)	0.195		−0.12	(−0.41 to 0.17)	0.431
B2		−0.31	(−0.63 to 0.01)	0.060		0.01	(−0.04 to 0.06)	0.714		−0.36	(−0.73 to −0.00)	0.049
C		0.10	(−1.04 to 1.24)	0.864		−0.07	(−0.23 to 0.10)	0.432		0.09	(−1.20 to 1.38)	0.892
Institution—THA type												
La Tour—standard		REF				REF				REF		
La Tour—custom		−0.04	(−0.56 to 0.47)	0.864		0.81	(0.74 to 0.89)	<0.001		−0.76	(−1.35 to −0.18)	0.011
USB—standard		−0.80	(−1.15 to −0.44)	<0.001		−0.24	(−0.29 to −0.19)	<0.001		−0.15	(−0.56 to 0.25)	0.453
LOS (days)		−0.03	(−0.12 to 0.06)	0.508		0.01	(−0.00 to 0.02)	0.090		−0.05	(−0.15 to 0.05)	0.354
Cementation type												
None		REF				REF				REF		
Hybrid **		−0.08	(−0.37 to 0.21)	0.595		−0.06	(−0.11 to −0.02)	0.003		0.13	(−0.20 to 0.46)	0.438
Fully cemented		−0.71	(−1.33 to −0.09)	0.025		0.00	(−0.09 to 0.09)	0.970		−0.83	(−1.53 to −0.12)	0.021
Year of operation												
2018		REF				REF				REF		
2019		−0.07	(−0.53 to 0.38)	0.749		0.01	(−0.06 to 0.08)	0.792		−0.04	(−0.56 to 0.48)	0.878
2020		−0.57	(−0.97 to −0.16)	0.006		−0.20	(−0.26 to −0.14)	<0.001		−0.27	(−0.73 to 0.19)	0.256
2021		−0.46	(−0.86 to −0.07)	0.022		−0.30	(−0.36 to −0.25)	<0.001		0.05	(−0.40 to 0.50)	0.822

PROM, patient-reported outcome measure; ASA, American Society of Anesthesiologists; LOS, length of stay; AC, Acetabulum; FE, Femur; REF, reference category; THA, total hip arthroplasty; β, regression coefficient; C.I., confidence intervals; *p*, *p*-value; *, for an increment of 10 units. ** AC uncemented and FE cemented. Note: analysis performed on 211 cases with complete data (13 cases were removed due to missing data).

## Data Availability

The raw data supporting the conclusions of this article will be made available by the authors on reasonable request.

## Supplementary Materials

The following supporting information can be downloaded at: https://www.mdpi.com/article/10.3390/jcm14103310/s1.

## Abbreviations

The following abbreviations are used in this manuscript:

THA	total hip arthroplasty
VBHC	value-based healthcare
PROM	patient-reported outcome measure
MCID	Linear dichroism
PASS	patient acceptable symptom state
OA	osteoarthritis
SCB	substantial clinical benefit
USB	University Hospital Basel
DRG	diagnosis-related group
CCER	Commission Cantonale d’Ethique de la Recherche
BMI	body mass index
ASA	American Society of Anesthesiologists
LOS	length of stay
mHHS	modified Harris Hip Score
pVAS	pain on a visual analogue scale
HOOS-PS	Hip disability and Osteoarthritis Outcome Score—Physical function Short form
CHF	Swiss francs
QQ	quantile–quantile
IQR	interquartile range
SD	standard deviation
VIF	Variance Inflation Factor
β	regression coefficient
EQ-5D	EuroQol 5-dimensions
COVID-19	COVID-19
OECD	Organisation for Economic Co-operation and Development
SIRIS	Swiss Implant Registry
