# Supplementary material for: Measuring Short-Term Outcomes Following Primary Total Hip Arthroplasty: A Value-Based Healthcare Approach
Source: J Clin Med. 2025 May 9;14(10):3310. doi: 10.3390/jcm14103310 (PMC12112243; doi:10.3390/jcm14103310)
Supplement: Supplementary file 1 [file jcm-14-03310-s001.zip › jcm-3551337-supplementary.pdf]

<u>Supplementary Material S1</u>: Number of hip replacement surgeries per 100 000 population performed in 2021 across OECD countries

Switzerland (323/100 000)
Germany (301/100 000)
Austria (287/100 000)
Finland (284/100 000)
Belgium (271/100 000)
Norway (255/100 000)
Netherlands (Not available)
France (246/100 000)
Denmark (236/100 000)
Iceland (223/100 000)
Ireland (222/100 000)
Sweden (215/100 000)
Luxembourg (207/100 000)
Czech Republic (198/100 000)
Australia (198/100 000)
Italy (194/100 000)
Lithuania (188/100 000)
Poland (183/100 000)
United Kingdom (180/100 000)
Slovenia (175/100 000)
Estonia (172/100 000)
Canada (153/100 000)
Croatia (152/100 000)
Latvia (152/100 000)
New Zealand (147/100 000)
Spain (129/100 000)
Bulgaria (128/100 000)
Portugal (108/100 000)
Slovak republic (99/100 000)
Hungary (79/100 000)
Israel (73/100 000)
Romania (65/100 000)
Korea (64/100 000)
Türkiye (55/100 000)
Chile (40/100 000)
Costa Rica (18/100 000)
Mexico (7/100 000)
